# Adherence to ketogenic diet in lifestyle interventions in adults with overweight or obesity and type 2 diabetes: a scoping review

**DOI:** 10.1038/s41387-023-00246-2

**Published:** 2023-09-14

**Authors:** Shiyu Li, Yan Du, Christiane Meireles, Kumar Sharma, Lu Qi, Alondra Castillo, Jing Wang

**Affiliations:** 1 School of Nursing, UT Health San Antonio, San Antonio, TX USA; 2 Center for Precision Medicine, Long School of Medicine, UT Health San Antonio, San Antonio, TX USA; 3 Department of Epidemiology, School of Public Health and Tropical Medicine, Tulane University, New Orleans, LA USA; 4 Department of Nutrition, Harvard T.H. Chan School of Public Health, Harvard University, Cambridge, MA USA; 5 College of Nursing, Florida State University, Tallahassee, FL USA

**Keywords:** Nutrition, Weight management

## Abstract

**Background/Objectives:**

Despite the evidence supporting the efficacy of the ketogenic diet (KD) on weight and type 2 diabetes (T2D) management, adherence to the KD is challenging. Additionally, no studies have reported changes in PA among individuals with overweight/obesity and T2D who have followed KD. We mapped out the methods used to assess adherence to the KD and level of physical activity (PA) in lifestyle interventions for weight and T2D management in individuals with overweight/obesity and T2D and compared levels of KD adherence and PA in these interventions.

**Methods:**

Articles published between January 2005 and March 2022 were searched in MEDLINE, CINAHL, and Scopus. Studies that included KD in lifestyle interventions for adults with T2D and overweight/obesity and measured ketone levels were included.

**Results:**

The eleven included studies comprised eight randomized controlled trials. They mainly used self-reported measures to evaluate adherence to the KD and level of PA. We found studies reported higher carbohydrate intake and lower fat intake than the KD regimen. Great inconsistencies were found among studies on the measurement and reporting of ketone and PA levels.

**Conclusion:**

Our results demonstrated the need to develop intervention strategies to improve adherence to the KD, as well as the necessity of developing standardized diet and PA assessment tools to establish a stronger evidence base for including KD in lifestyle interventions for weight and T2D management among adults with overweight/obesity and T2D.

## Introduction

Diabetes is a significant public health concern. The projected prevalence of type 2 diabetes (T2D) is expected to rise from 6.28% in 2017 to 10.9% by 2045 [1]. T2D significantly increases the risk of diabetes-specific complications and mortality, making it the tenth leading cause of mortality globally and placing a substantial economic burden on individuals living with T2D and the healthcare system [2, 3]. For example, in 2019, direct health expenditure on diabetes was estimated at 760 billion USD, and this cost is expected to increase to 845 billion USD by 2045 [4]. Therefore, implementing cost-effective strategies is crucial to mitigate the burden of diabetes and T2D.

Lifestyle interventions including diet, physical activity (PA), and weight management components have been recommended by American Diabetes Association (ADA) as the frontline treatment strategy for people living with T2D [5]. Diet is an important component in lifestyle intervention. A meta-analysis demonstrated that despite the type of diet, most diet trials have reported modest weight loss after 12 months [6]. Traditionally, low-fat diet has been utilized in large randomized controlled trials (RCTs) for achieving weight loss and managing T2D [7]. For example, in the landmark Look AHEAD trial, participants followed a low-fat low-calorie diet as part of a lifestyle intervention for adults with overweight/obesity and T2D, and >50% of participants achieved and maintained at least 5% weight loss over 8 years [8]. Despite the various dietary patterns available for weight and T2D management, the ideal diet pattern is not yet conclusive [7].

The ketogenic diet (KD) is a very low-carbohydrate diet that restricts daily carbohydrate intake to ~20–50 g/d or >10% of total daily caloric intake [9], with 70–80% calories derived from fat. By restricting carbohydrate intake, KD aims to induce a state of nutritional ketosis. During ketosis, the liver typically consumes fat to produce and secrete ketones and fatty acids as the primary energy source for body tissues [10, 11]. Emerging evidence suggests that KD can be considered as first-line treatment approach for obesity management due to its ability to suppress hunger, reduce lipogenesis, increase lipolysis, enhance metabolic efficiency for fat utilization, and boost energy expenditure [12, 13]. Research also indicates that KD can be a first-line approach for T2D treatment [13]. Given that T2D is characterized by hyperglycemia and that dietary carbohydrate intake has a significant impact on glycemic levels, reducing carbohydrate intake is a reasonable strategy for effective T2D management. Studies have consistently demonstrated that KD positively influences glycemic control in individuals with T2D by reducing glucose uptake from dietary carbohydrates, improving systemic insulin sensitivity, reducing insulin requirements, and facilitating weight loss [12].

Recent meta-analyses of randomized controlled trials consistently demonstrated the efficacy of KD for individuals with overweight/obesity and T2D compared to usual diet or other recommended diets for diabetes management [14–16]. For example, Choi et al. compared the efficacy of KD to low-fat diets KD and found that KD was more effective than low-fat diets in promoting weight loss and glycemic control [14]. Moreover, short-term KD interventions (≤6 months) have been found to result in greater weight loss, improved glycemic control, reduced diabetes medication usage, and better lipid profile compared to other recommended diets for individuals with overweight/obesity and T2D [15]. Therefore, the KD may be a viable option for enhancing the efficacy and effectiveness of lifestyle interventions for weight and T2D management.

The efficacy of KD for adults with overweight/obesity and T2D can be limited by the declined adherence to the diet. Studies have found that participants who had higher levels of adherence to the KD achieved better weight loss outcomes compared to those with lower adherence [15, 17]. However, while studies consistently reported adhering to KD was challenging, no previous reviews have examined or compared the level of KD adherence among participants with overweight/obesity and T2D [15, 17–19]. Although one narrative review reported KD adherence levels in various KD interventions, it did not systematically compare the differences in macronutrient intakes across studies [20]. Given that meeting macronutrient recommendations are important for achieving ketosis, comparing macronutrient intakes in various KD interventions can help to facilitate improvement in nutrition strategies and maximize the effectiveness of the KD [21]. Therefore, we aimed to investigate adherence to the KD and levels of PA in lifestyle interventions for adults with overweight/obesity and T2D. Specifically, this study aimed to map out the methods used to evaluate adherence to the KD and PA levels and to compare macronutrient proportions in KD groups.

## Methods

This scoping review was conducted following the modified Arksey & O’Malley’s 5-step framework and reported following the PRISMA-ScR guidelines [22, 23].

### Identify research question

This review was guided by the following questions: What are the assessment methods employed in previous lifestyle interventions to evaluate adherence to the KD and PA among persons living with T2D and overweight/obesity? What are the levels of adherence to the KD and PA among these individuals during the intervention? Scoping reviews aim to “map the literature on a particular topic” [23]. Given the breadth of the present study’s research question and the heterogeneity among existing research, we selected a scoping review approach [24].

### Identify relevant studies

The following procedures were performed to identify relevant studies: (1) An initial search in the MEDLINE database was performed using the terms “Ketogenic Diet” and “Type 2 Diabetes” to identify keywords and form a comprehensive search strategy (Appendix 1); (2) A second search incorporating all identified keywords and Medical Subject Headings were conducted across MEDLINE, Scopus, and CINAHL to retrieve literature published between January 2005 and September 2021; (3) An updated search using the same procedure was conducted in March 2022 to identify any newly published interventional studies. Six review articles on the effect of KD on persons living with T2D were manually searched to identify any interventional studies that had not been included [12, 15–18, 25]. An experienced librarian conducted the initial search and supervised the subsequent procedures.

### Study selection

The inclusion criteria for this study were as follows: (1) interventional studies including randomized and non-randomized controlled trials, quasi-experimental designs, and before-and-after studies; (2) published between January 2005 and March 2022; (3) written in English; (4) examining the effect of self-prepared KD with free food choices as part of a lifestyle intervention; (5) included adults with overweight/obesity (age ≥18 years, BMI ≥ 25 kg/m^2^) (6) included persons living with T2D. A KD was defined as having <10% of daily total caloric intake from carbohydrates, or a daily carbohydrate intake of 20–50 grams (g) [26]. Lifestyle intervention was defined as a multi-component program comprising at least two components from diet, exercise, or behavior change strategies [27]. Studies were excluded if they met any of the following criteria: (1) being a case study, systematic review, meta-analysis, protocol description, conference abstract, or news article; (2) having a cross-over design; (3) not monitoring ketone levels, (4) being a feeding trial.

The search results were imported into the Endnote (Clarivate Plc, London, United Kingdom) to remove duplicate citations, and screened for title and abstracts through Rayyan (Rayyan Systems Inc, Cambridge, MA), an online literature review software. Two reviewers independently assessed the titles and abstracts in Rayyan using the aforementioned criteria. All studies meeting the eligibility criteria were retrieved for full-text screening. Any discrepancies in the inclusion or exclusion of studies were resolved through discussion with a third reviewer.

### Data charting

Data was extracted from eligible studies using a data extraction form (Table 1) that was created in Microsoft Excel (Microsoft Cooperation LLC, Redmond, WA, USA) and piloted by the first author (SL) prior to data extraction (Table 1). Two researchers (SL and AC) performed data extraction using the form and the extracted data was then checked for accuracy and completeness by SL. Studies that did not meet the eligibility criteria were excluded during this phase.

**Table 1:** Key information extracted from included studies.

Study characteristics	a. Title
	b. Author
	c. Year
	d. Country
	e. Study settings
	f. Study design
	g. Duration
	h. Inclusion/exclusion criteria
	i. Participants characteristics (sample size, age, sex, race/ethnicity, baseline BMI, baseline HbA1c, retention rate)
Intervention characteristics	a. KD and control diet recommendations (if applicable)
	b. Physical activity recommendations
	c. Behavior change strategies
Diet adherence measures and outcomes	a. Diet adherence measures
	b. Self-reported diet adherence outcomes
	c. Ketone outcomes
Physical activity measures and outcomes	a. Physical activity measures
	b. Physical activity outcomes
Weight and glycemic control outcomes	a. Weight outcomes
	b. Glycemic control outcomes

### Collating, summarizing, and reporting

The findings of this scoping review were summarized and reported in two ways, in line with our two research questions. We provided a narrative summary of the assessment methods used to evaluate adherence to KD and levels of physical activity levels. We also provided a descriptive numerical summary of the levels of adherence to the KD and physical activity.

## Results

### Study characteristics

**Fig. 1:**
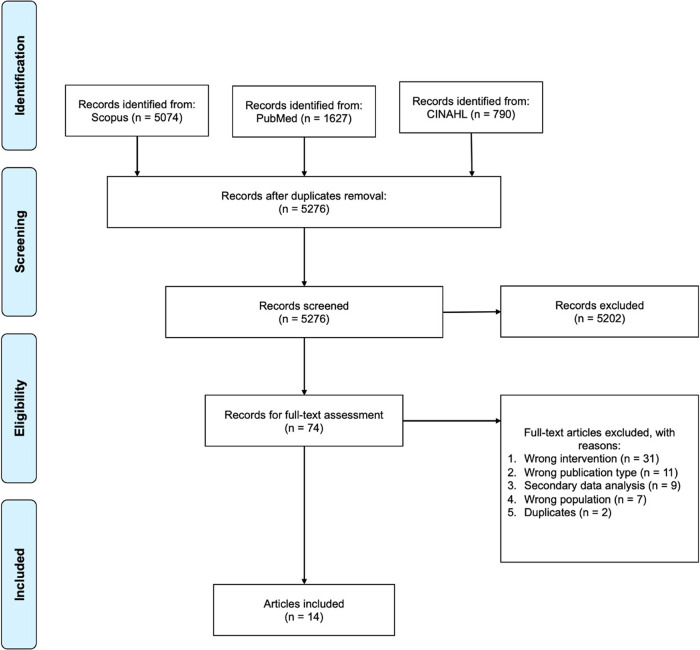
PRISMA flow diagram of study selection.

The total number of articles retrieved through the initial electronic search were 7491. After removing duplicates and screening abstracts, 74 articles remained to be read in full. Based on selection criteria, a total of 14 articles representing eleven different trials were included in this review (Fig. 1).

Table 2 presents the characteristics of the included studies. We included eight RCTs [28–37], one non-RCT [38, 39], and two studies with a single-arm pre-post design [40, 41]. All included studies specified inclusion of individuals with BMI ≥ 25 kg/m^2^. Definition for T2D varied based on criteria such as elevated glycemic control (using different HbA1c thresholds) or a clinical diagnosis. Inclusion criteria regarding T2D medication varied, with some studies excluding individuals using insulin or other T2D medication, while others did not specify T2D medication requirements. The total number of participants following a KD was 689, with the mean age ranging from 38.3 ± 26 [40] to 64.8 ± 7.7 [33] years. Only studies conducted in the US provided race/ethnicity data, and the majority of these studies predominantly consisted of Caucasian or White participants. The mean body mass index (BMI) of participants ranged from 32.2 ± 6.3 [37] to 40.4 ± 8.8 [39] kg/m^2^, and the mean baseline A1c ranged from 6.3 ± 1.1 [29] to 8.9 ± 0.4% [40]. As shown in Table 3, the duration of included studies ranged from 90 days [40] to 2 years [36, 38], with the retention rate of KD group ranging from 43.8% [28] to 100% [40]. All studies recommended a self-prepared KD with no restrictions on food choices and included physical activity as part of the intervention, along with additional components such as nutrition counseling [30, 32–34, 37–40], monitoring [30, 38–40], feedback [30, 41], and group education [28, 31, 35, 36]. Of the nine studies that had a control group, five compared the KD intervention to the typical diabetes lifestyle education recommended by T2D management guidelines [30, 31, 33, 34, 39]. Three studies compared the KD to other dietary patterns that had different energy and macronutrient goals [28, 29, 35, 36], and one study compared the combination of KD education and mindfulness training to the KD education only [37].

**Table 2:** Participant characteristics across included studies.

Author	Year	Country	Participant characteristics		T2D definition	Diabetes medication
			KD	Control		
Yancy [38]	2005	USA	*N* = 28 Age: 56.0 (7.9) Female: 1 (5%) White: 13 (62%) Black or African American: 8 (38%) T2D: 100% Insulin Treatment: 5 (17.9%) Baseline BMI: 42.2 (5.8) Baseline A1c: 7.5 (1.4)		Elevated HbA1c (>6.5%) without medications, or treatment with oral hypoglycemic agents (OHA) and/or insulin.	Inclusion criteria: treatment with oral hypoglycemic agents (OHA) and/or insulin.
Westman [25]	2008	USA	*N* = 48 Age: 51.8 (7.3) Female: 76.3% White: 57.9% Black or African American: 36.8% T2D: 100% Insulin Treatment: 8 (38.1%) Baseline BMI: 37.7 (6.1) Baseline A1c: 8.8 (1.8)	*N* = 49 Age: 51.8 ± 7.8 Female: 80.4% White: 45.7% Black or African American: 50% T2D: 100% Insulin Treatment: 3 (10.3%) Baseline BMI: 38.5 ± 5.6 Baseline A1c: 8.3 ± 1.9	Diagnosis of T2D for more than 1 year, confirmed by HbA1c > 6.0%	No eligibility criteria on medication intake
Yancy [26]	2010	USA	*N* = 72 Age: 52.9 (10.2) Female: 20 (28%) White: 31 (43%) T2D: 22 (31%) Baseline BMI: 39.9 (6.9) Baseline A1c: 6.3 (1.1)	*N* = 74 Age: 52.0 (9.2) Female: 21 (28%) White: 31 (42%) T2D: 24 (32%) Baseline BMI: 38.8 (7.0) Baseline A1c: 6.4 (1.3)	No eligibility criteria on T2D diagnosis, but HbA1c ≤ 11% at screening	No eligibility criteria on medication intake
Goldstein [28]	2011	Israel	*N* = 26 Age: 57 (9.0) Female: 13 (50%) T2D: 100% Baseline BMI: 33.1 (3.6) Baseline A1c: 9.0 (1.7)	*N* = 26 Age: 55 (8.0) Female: 14 (53.8%) T2D: 100% Baseline BMI: 33.3 (3.0) Baseline A1c: 8.8 (1.2)	elevated HbA1c (>7%)	Exclusion criteria: insulin treatment
Saslow [29, 30]	2014, 2017	USA	*N* = 16 Age: 64.8 (7.7) Female: 9 (56.3%) White: 13 (81.3%) Black or African American: 1 (6.3%) Hispanic/Latino: 1 (6.3%) Asian/Pacific Islander: 1 (6.3%) T2D: 9 (56.3%) Baseline BMI: 36.2 (8.2) Baseline A1c: 6.6 (0.3)	*N* = 18 Age: 55.1 (13.5) Female: 16 (88.9%) White: 11 (61.1%) Black or African American: 1 (5.6%) Hispanic/Latino: 2 (11.1%) Asian/Pacific Islander: 4 (22.2%) T2D: 13 (72.2%) Baseline BMI: 36.2 (8.2) Baseline A1c: 6.6 (0.3)	diagnosis of T2D (HbA1c ≥ 6.5%) or prediabetes (HbA1c > 6.0%)	Exclusion criteria: currently using insulin or taking more than three oral hypoglycemic medications
Tay [32, 33]	2015, 2018	Australia	*N* = 58 Age: 58 (7) Female: 21 (36) T2D: 100% Insulin Treatment: 6 (10%) Baseline BMI: 34.2 (4.5) Baseline A1c: 7.3 (1.1)	*N* = 57 Age: 58 (7) Female: 28 (49) T2D: 100% Insulin Treatment: 6 (11%) Baseline BMI: 35.1 (4.1) Baseline A1c: 7.4 (1.1)	HbA1c ≥ 7.0% or taking a diabetes medication	Inclusion criteria: taking a diabetes medication
Saslow [31]	2017	USA	*N* = 12 Age: 53.0 (10.2) Female: 50% White: 7 (58%) Asian/Pacific Islander: 2 (17%) Black/African American: 3 (25%) Latino/a: 2 (17%) T2D: 100% Baseline A1c: 7.1 (0.4)	*N* = 13 Age: 58.2 (6.7) Female: 69% White: 8 (62%) Asian/Pacific Islander: 2 (15%) Black/African American: 0 Latino/a: 5 (38%) T2D: 100% Baseline A1c: 7.2 (0.3)	HbA1c between 6.5–9% at screening	Exclusion criteria: use of any diabetes medication other than metformin
Hallberg [36] Athinarayanan [35]	2018, 2020	USA	*N* = 262 Age: 53.8 (8.4) Female: 66.8% T2D: 100% Insulin Treatment: 46% Baseline BMI: 40.4 (8.8) Baseline A1c: 7.6 (1.5)	*N* = 87 Age: 52.3 (9.5) Female: 58.6% T2D: 100% Insulin Treatment: 30% Baseline BMI: 36.7 (7.3) Baseline A1c: 7.6 (1.8)	Diagnosis of T2D	No eligibility criteria on medication intake
Mason [34]	2019	USA	*N* = 58 Age: 58.7 (10.9) Female: 63.8% White: 29 (58%) Black: 10.3% Latino/Hispanic: 10.3% Asian/Pacific Islander: 20.7% Other: 8.6% T2D: 100% Insulin Treatment: 6 (11.5%) (completers only) Baseline BMI: 32.2 (6.3) Baseline A1c: 7.7 (1.3)		Diagnosis of T2D and confirmed by 6.5% ≤ HbA1c < 12.0% at screening	No eligibility criteria on medication intake Insulin: *N* = 6
Walton [37]	2019	USA	*N* = 11 Age: 38.3 (26) Female: 11 (100%) White: 11 (100%) T2D: 100% Baseline BMI: 36.3 (1.4) Baseline A1c: 8.9 (0.4)		Recent diagnosis of type 2 diabetes mellitus based on HbA1c of 6.5% or higher	Exclusion criteria: use of any diabetes medication
Durrer [27]	2020	Canada	*N* = 98 Age: 58 (11) Female: 56% T2D: 100% Baseline BMI: 36.0 (6.0) Baseline A1c: 7.9 (1.5)	*N* = 90 Age: 59 (8) Female: 57% T2D: 100% Baseline BMI: 35.1(5.3) Baseline A1c: 7.8 (1.4)	Diagnosis of T2D	Inclusion criteria: use of at least one glucose-lowering medication

**Table 3:** Intervention characteristics across included studies.

Author	Design, duration	Retention rate		Diet recommendations		PA recommendations	
		KD	Control	KD	Control	KD	Control
Yancy [38]	Single-arm, 16 weeks/4 months	Week 16: 21/28 (75%)		**Low-carbohydrate, ketogenic diet** Carbs: <20 g/d Protein: no restriction Fat: no restriction Energy: no restriction		Encouraged aerobic exercise, 30 min * 3×/week	
Westman [25]	RCT, 24 weeks	Week 24: 21/48 (43.8%)	Week 24: 29/49 (59.2%)	**Low-carbohydrate, ketogenic diet:** Carbs: <20 g/d Protein: no restriction Fat: no restriction Energy: no restriction	**Low-glycemic index diet:** Carbs: 55% of daily caloric intake Protein: not mentioned Fat: not mentioned Energy intake: 500 kcal/d less than a participant’s calculated energy intake for weight maintenance	Encouraged exercise, 30 min * 3×/week	Same as intervention group.
Yancy [26]	RCT, 48 weeks	Week 48: 57/72 (79.2%)	Week 48: 65/74 (87.8%)	**Low-carbohydrate diet:** Carbs: <20 g/d; when approached weight loss goals or cravings threatened diet adherence, gradually add ~5 g/d each week until weight was maintained, or cravings diminished. Protein: no restriction Fat: no restriction Energy: no restriction	**Orlistat plus low-fat diet:** Carbs: not mentioned Protein: not mentioned Fat: <30% daily caloric intake - Saturated fat: <10% daily caloric intake - Cholesterol: <300 mg/d Energy: 500–1000 kcal/d below a participant’s calculated energy intake for weight maintenance Orlistat: a 30-day supply of orlistat (120 mg before meals 3 times a day) was provided monthly	Encouraged exercise, 30 min * 3×/week	Same as intervention group.
Goldstein [28]	RCT, 12 months	6 months: 20/26 (76.9%) 12 months: 14/26 (53.8%)	6 months: 20/26 (76.9%) 12 months: 16/26 (61.5%)	**Modified atkins diet:** Carbs: up to 25 g/d for the first 6 weeks and increase to up to 40 g/d afterwards. Protein: No restriction Fat: No restriction Energy: No restriction	**ADA-diet (2001 version):** Carbs: 80% daily caloric intake divided between carbs and fats - Fiber: 35 g/d Protein: 10–20% daily caloric intake Fat: - MUFA: 18–20% - PUFA: 8–10% - SFA: 9–10% Energy: - Men: ≤1500 kcal/d - Women: ≤1200 kcal/d	Encouraged exercise, 3×/week	Same as intervention group.
Saslow [29, 30]	RCT, 12 months	3 months: 15/16 (93.8%) 12 months: 14/16 (87.5%)	3 months: 18/18 (100%) 12 months: 15/18 (83.3%)	**Low-carbohydrate, ketogenic diet:** Carbs: 20–50 g/d net carbohydrate Protein: normal amount to meet IOM suggested minimum amount Fat: no restriction Energy: no restriction	**Moderate-carbohydrate, calorie-restricted diet (ADA guidelines)** Carbs: 45–50% daily caloric intake Protein: keep as usual Fat: lower fat consumption Energy intake: 500 kcal/d fewer than their calculated weight maintenance needs	Provided PA education	Same as intervention group.
Tay [32, 33]	RCT, 2 years	6 months: 46/58 (79.3%) 1 year: 41/58 (70.7%) 2 years: 33/58 (56.9%)	6 months: 47/57 (82.5%) 1 year: 37/57 (64.9%) 2 years: 28/57 (49.1%)	**Hypocaloric low-carbohydrate diet:** Carbs: 14% daily caloric intake (<50 g/d) Protein: 28% daily caloric intake Fat: 58% daily caloric intake - 35% MUFA - 13% PUFA - <10% SFA Energy: 500–1000 kcal/d caloric restriction	**Hypocaloric high-carbohydrate diet:** Carbs: 53% daily caloric intake - Emphasize low-glycemic index foods Protein: 17% daily caloric intake Fat: <30% daily caloric intake - 15% MUFA - 9% PUFA - <10% SFA Energy intake: 500–1000 kcal/d caloric restriction	Provided supervised aerobic and resistence exercise sessions, 60 min * 3×/week	Same as intervention group.
Saslow [31]	RCT, 32 weeks	16 weeks: 12/12 (100%) 32 weeks: 11/13 (92%)	16 weeks: 8/13 (61.5%) 32 weeks: 7/13 (54%)	**Very-low-carbohydrate ketogenic diet:** Carbs: 20–50 g/d net carbohydrate Protein: not mentioned Fat: Not mentioned Energy: no restriction	**Create your plate diet (ADA recommendations):** Carbs: green vegetables and somewhat limited starchy and sweet foods Protein: lean protein sources Fat: emphasize low-fat Energy: not mentioned Others: All proportions are based on a 9-inch plate:	Provided PA education	Same as intervention group.
Hallberg [36] Athinarayanan [35]	open-label, non-randomized, controlled, before-and-after study design, 2 years	1 year: 218/262 (83.2%) 2 years: 194/262 (74%)	1 year: 78/87 (89.7%) 2 years: 68/87 (78%)	**Carbohydrate-restricted diet to achieve nutritional ketosis:** Carbs: <30 g/d Protein: 1.5 g/kg of ideal body weight; adjusted as necessary Fat: to satiety, no restriction Energy: no restriction	**ADA nutritional and lifestyle recommendations (2015 to 2018):** Carbs: not mentioned Protein: not mentioned Fat: not mentioned Energy: not mentioned	Provided PA education	Follow ADA Lifestyle Recommendations
Mason [34]	RCT (both groups on KD diet), 6 months	6 months: 55/58 (94.8%)		**Carbohydrate-restricted diet:** Carbs: 20–35 g/d net carbs; up to 50 g/d net carbs Protein: adequate amount as described by the IOM Fat: to satiety Energy: no restriction	Same as intervention	Provided PA education	Same as intervention
Walton [37]	Single-arm, 90 days	90 days: 11/11 (100%)		**Low-carbohydrate, high-fat diet:** Carbs: ~5% daily caloric intake, up to 30 g/d Protein: 20–25% daily caloric intake Fat: 70–75% daily caloric intake Energy intake: not mentioned		Encouraged to continue pre-existing physical activities	
Durrer [27]	RCT, 12 weeks	12 weeks: 78/98 (79.6%)	12 weeks: 60/90 (66.7%)	**Low-carbohydrate, energy-restricted diet:** Carbs: <50 g/d Protein: 110–120 g/d Fat: 35–45 g/d Energy: 850–1100 kcal/d	**Diet and lifestyle recommendations from canadian diabetes association:** Carbs: not mentioned Protein: not mentioned Fat: not mentioned Energy: not mentioned	Not reported	Follow lifestyle recommendations from Canadian Diabetes Association

Table 4 presents changes in weight loss and diabetes-related outcomes across included studies. All studies found significant weight loss (3.4 [31] to 12.7 kg [34]) within KD groups. Six of them showed that the KD groups lost significantly more weight than the control groups [28, 30, 32, 34, 38, 39], while two studies found no differences in weight loss between KD and control groups after 1 year to 2 years [31, 36]. Out of eight studies that measured levels of A1c, seven reported a significant decrease in A1C levels (0.3 [29] to 3.3% [40]) in KD groups [28, 29, 33, 36, 38, 40, 41], with five demonstrating greater A1c reduction in the KD groups [28, 30, 32, 34, 38] and three reporting no difference [29, 31, 36]. All but two studies measured diabetes-related health outcomes including fasting blood glucose, fasting insulin, insulin resistance, and/or others. Among these studies, five reported significant improvements in at least one diabetes-related health outcome within the KD group [29, 30, 35, 36, 38, 39, 41]. Changes in diabetes medication across included studies were also presented in Table 4.

**Table 4:** Changes in weight loss and diabetes-related outcomes across included studies.

Author	Year	Duration	Changes in weight and HbA1c			Main findings on glycemic control	Changes in diabetes medication	
			KD	Control	Findings		Intervention	Control
Yancy [38]	2005	12 weeks	Weight: −8.7 kg* HbA1c: −1.2%*			***Fasting glucose:*** significant reduction in KD group at 16 weeks.	***Diabetes medication (all)*** - Reduced: 10/21 (47.6%) - Discontinued: 7/21 (33.3%) - Unchanged: 4/21 (19.0%)	
Westman [25]	2008	24 weeks	Weight: −11.1 kg* HbA1c: −1.5*	Weight: −6.9 kg* HbA1c: −0.5	Weight: Favors KD HbA1c: Favors KD	***Fasting insulin:*** similar significant reduction in both groups at 24 weeks.	***Diabetes medication (all)*** - Discontinued or reduced: 20/21 (95.2%) ***Insulin:*** ***-*** Discontinued: 4	***Diabetes medication (all)*** - Discontinued or reduced medication: 18/29 (62.1%) ***Insulin:*** - Discontinued: 1
Yancy [26]	2010	48 weeks	Weight: −11.37 kg* HbA1c: −0.30%*	Weight: −9.62 kg* HbA1c: −0.06%, N.S.	Weight: Favors KD HbA1c: No difference	***Fasting glucose:*** significant reduction in KD group at 48 weeks; ***Fasting insulin:*** significant reduction in fasting insulin level in KD group participants (without diabetes and were not taking diabetes medications only).	***Diabetes medication (all)*** - Reduced: 13/16 (81%) - Increased: 1/16 (6%)	***Diabetes medication (all)*** - Reduced: 15/22 (68%) - Increased: 1/22 (5%)
Goldstein [28]	2011	12 months	Weight: −3.4 kg* HbA1c: −1%, N.S.	Weight: −5.4 kg* HbA1c: −1%*	Weight: No difference HbA1c: No difference	***Fasting glucose:*** no significant differences between groups over time.	***Diabetes medication (all)*** - Reduced: 17/26 at 3 months	***Diabetes medication (all)*** - Reduced: 11/26 at 3 months
Saslow [29, 30]	2014, 2017	12 months	Weight: −7.9 kg HbA1c: −0.5%	Weight: −1.7 kg HbA1c: 0	Weight: Favors KD HbA1c: Favors KD	***Fasting glucose:*** no significant change within each diet group and no significant difference between diet groups over time ***Fasting insulin:*** no significant change within each diet group and no significant difference between diet groups over time ***HOMA2-IR:*** no significant change within each diet group and no significant difference between diet groups over time	***Oral diabetes medication:*** - Discontinued (one or more): 7/16 (44%) at 3 months ***Sulfonylureas or dipeptidyl peptidase-4 inhibitors:*** - Discontinued: *n* = 6 at 12 months ***Metformin:*** - Discontinued: *n* = 3 at 12 months - Increased: *n* = 1 at 12 months	***Oral diabetes medication:*** - Discontinued (one or more): 2/18 (11%) at 3 months ***Sulfonylureas or dipeptidyl peptidase-4 inhibitors:*** - Discontinued: *n* = 0 at 12 months ***Metformin:*** - Discontinued: *n* = 2 at 12 months - Increased: *n* = 0 at 12 months
Tay [32, 33]	2015, 2018	2 years	Weight: −6.8 kg* HbA1c: −0.6%*	Weight: −6.6 kg* HbA1c: −0.9%*	Weight: No difference HbA1c: No difference	***Fasting glucose:*** similar significant reduction in both groups at 1 and 2 years; ***Fasting insulin:*** similar significant reduction in both groups at 1 and 2 years; ***HOMR-IR, HOMA-%B:*** similar significant reduction in both groups at 1 and 2 year ***Glycemic variability indexes:*** significant more reduction in KD compared to control at 1 and 2 years ***Proportion of time in hyperglycemic range:*** Significantly lower proportion of time for KD group ***Proportion of time in hypoglycemic range:*** no difference between diet groups	***Medication effect score:*** - Reduced: *n* = 22 - Greater reduction in MES in KD versus control group	***Medication effect score:*** - Reduced: *n* = 9
Saslow [31]	2017	32 weeks	Weight: −12.7 kg* HbA1c: −0.8%*	Weight: −3 kg HbA1c: −0.3%*	Weight: Favors KD HbA1c: Favors KD	***Diabetes-related distress:*** no significant differences between groups at 16 or 32 weeks.	***Metformin:*** - Decreased: *n* = 1 - Increased: *n* = 2 - Unchanged: *n* = 8	***Metformin:*** - Decreased: *n* = 2 - Increased: *n* = 1 - Unchanged: *n* = 4
Hallberg [36] Athinarayanan [35]	2018, 2020	2 years	Weight: −11.94 kg* HbA1c: −0.9%*	Weight: −1.28 kg HbA1c: −0.4%*	Weight: Favors KD HbA1c: Favors KD	***Fasting glucose:*** significant reduction in KD group versus control group at 1 and 2 years; ***Fasting insulin:*** Significant reduction in KD group versus control group at 1 and 2 years ***HOMA-IR (derived from fasting insulin):*** Significant reduction in KD group versus control group at 1 and 2 years (excluding exogenous users); ***Diabetes reversal:*** 53.5% in KD group versus 0% in the control group at 2 years; ***Diabetes remission (partial or Complete):*** 17.6% in KD group versus 2.4% in the control group at 2 years; ***Complete diabetes remission:*** 6.7% in KD group versus 0% in the control group at 2 years;	***Diabetes medication (all)*** - Significant reduction in KD versus low-fat group at 1 year ***Insulin dose*:** - Significant reduction in KD versus low-fat group at 1 and 2 years - Mean dose: 15.5 U/day at 2 year	***Insulin dose:*** - No change in mean insulin dose - Mean dose: 109.3 U/day at 2 year
Mason [34]	2019	6 months	N.R.					
Walton [37]	2019	90 days	Weight: −9 kg* HbA1c: −3.3%*	N.R.				
Durrer [27]	2020	12 weeks	Weight: −10.4 kg HbA1c: −1.9%	Weight: + 0.5 kg HbA1c: 0	Weight: Favors KD HbA1c: Favors KD	***Fasting glucose:*** significantly more reduction in KD versus control group at 12 weeks.	***Glucose-lowering medication (all):*** - Discontinued: 35.7% ***Medication effect scale:*** - significantly lower mean MES score in KD than control group - mean score: 0.6	***Glucose-lowering medication (all):*** - Discontinued: 0% ***Medication effect scale:*** - mean score: 2.2

### Diet and physical activity measures

As shown in Table 3, dietary adherence measures used in the studies included self-reports (*n* = 9) [28–37, 41] and biomarkers such as ketone levels (*n* = 10) [28–41] and 24-hour urinary-urea/creatinine ratio (*n* = 1) [36]. Formats of self-reported dietary adherence included take-home food records (*n* = 5) [28–31, 41], electronic food logs (*n* = 2) [33, 36], 24-hour dietary recall (*n* = 2) [32, 33, 37], and self-rated diet adherence on a Likert Scale (*n* = 1) [31]. Studies measured ketone levels for the purpose of indicating diet adherence at follow-ups (*n* = 9) and encouraging participants to better adhere to KD as a behavior change strategy (*n* = 5). However, studies varied on measurement target and timeframes, with some studies measuring urine ketone levels (*n* = 5), and others blood beta-hydroxybutyrate (BHB) levels (*n* = 6) at varying frequencies ranging from daily to yearly [30, 33, 35, 37, 39, 40]. Of the six studies evaluating physical activity levels [28, 29, 31, 32, 34–36], 4 utilized standardized questionnaires, 1 relied on questions to report daily exercise sessions [28], and 1 objectively assessed physical activity levels based on the 7-day accelerometry [35].

### Intervention effect on energy intake

Two studies limited daily caloric intake in the KD group, while daily caloric intake goals varied in the control group across studies [30, 36] (Table 3). Specifically, four studies advised the control group to limit daily caloric intake to 500–1000 kcal below the recommended amount for weight maintenance [28, 29, 33, 35], three recommended following lifestyle guidelines for T2D management [30, 34, 39], and one study recommended men to consume ≤1500 kcal/d and women ≤1200 kcal/d [31].

In the 6 studies without caloric restrictions in the KD groups [28, 29, 31, 33, 34, 41], all reported a decrease in daily caloric intake, ranging from 419.3 kcal/d after one year [32] to 687 kcal/d after 48 weeks [29]. The final daily caloric intake in the KD groups was between 1329.2 kcal/d at 32 weeks [34] to 1725 kcal/d at 1 year [31]. Out of the seven studies with a control group, six showed a reduction in daily caloric intake [28–32, 34], from 139 kcal/d after 12 weeks [30] to 793 kcal/d after 24 weeks [28]. The final daily caloric intake ranged from 1335 kcal after 24 weeks [28] to 1937 kcal after 1 year [31]. One study reported a 207 kcal/d increase in daily caloric intake despite recommending a low-calorie diet [36]. The differences in daily calorie intake between KD and control groups are presented in Table 5.

**Table 5:** Changes in daily caloric intake and macronutrient consumption across included studies.

Author	Timepoint	Changes in daily caloric intake			Changes in carbohydrate intake			Changes in protein intake			Changes in fat intake		
		KD	Control	KD vs. control	KD	Control	KD VS. control	Intervention	Control	KD vs. C	Intervention	Control	I vs. C
Yancy [38]	16 weeks	−612.8^a^	N.A.	N.A.	Total carbs (g/d): −170.6^a^	N.A.	N.A.	Protein (g/d): −2.7^a^	N.A.	N.A.	Fat (g/d): −2^a^	N.A.	N.A.
Westman [25]	24 weeks	−578^a^	−793^a^	N.R.	Total carbs (g/d): −196^a^	Total carbs (g/d): −96^a^	N.R.	Protein (g/d): +22^a^	Protein (g/d): −19^a^	N.R.	Fat (g/d): +13	Fat (g/d): −33	N.R.
Yancy [26]	48 weeks	−687^a^	−618^a^	No difference	Total carbs (g/d): −200^a^	Total carbs: −36.6^a^	KD < C	Protein (g/d): +16.7^a^	Protein (g/d): −19.1^a^	N.R.	Fat (g/d): +1.8^a^	Fat (g/d): −43^a^	N.R.
Goldstein [28]	1 year	−536^*^	−662^*^	No difference	Total carbs (g/d): −128^*^	Total carbs (g/d): −40^*^	KD < C	Protein (g/d): −3	Protein (g/d): −29^*^	KD < C	Fat (g/d): −1	Fat (g/d): −43^*^	KD > C
Saslow [29, 30]	1 year	−419.3^a^	−382^*^	No difference	Net carbs (g/d): −102.5^*^	Net carbs (g/d): −34.6^*^	KD < C	Protein (g/d): +14.9^a^	Protein (g/d): −22.6^a^	KD > C	Fat (g/d): +26.2	Fat (g/d): −10.9	KD > C
Tay [32, 33]	2 years	N.R.	N.R.	No difference	N.A.	N.A.	KD < C	N.R.	N.R.	KD > C	N.A.	N.A.	KD > C
Saslow [31]	32 weeks	−439.3^a^	−216.6^a^	No difference	Net carbs (g/d): −122.7^*^	Net carbs (g/d): −14.8	KD < C	Protein (g/d): −1.6	Protein (g/d): −0.1	No difference	Fat (g/d): +4	Fat (g/d): + 23.7	No difference
Mason [34]	6 months	N.R.	N.R.	N.R.	Net carbs: −111.45^a^			N.R.			N.R.		
Durrer [27]	12 weeks	805^a^	−139^a^	N.R.	Total carbs (g/d): −200^a^	Total carbs (g/d): +26^a^	N.R.	Protein (g/d): +21^a^	Protein (g/d): −3^a^	N.R.	Fat (g/d): −38^a^	Fat (g/d): −2^a^	N.R.

^a^Calculated by the authors, *p < 0.05; *NR* not reported, *NA* not applicable, *KD* ketogenic diet group, *C* control group.

### Intervention effect on carbohydrate intake

All included studies restricted daily carbohydrate intake in KD groups to less than 50 g/d. In two of the studies [29, 31], participants were allowed to gradually increase their daily carbohydrate intake (Table 3). For example, Yancy et al. advised the participants to gradually increase their daily carbohydrate intake by ~5 g/d once the weight loss goals were reached or if cravings posed a threat to diet adherence [29]. Goldstein et al. restricted daily carbohydrate intake to less than 25 g/d during the first 6 weeks and then increased it up to 40 g/d [31]. Of the 8 studies that included a control group other than KD, 3 of them suggested a moderate daily intake of carbohydrates that constituted between 45 to 53% of the total caloric intake [28, 33, 35]. Four other studies recommended that the control group follow a diet recommended for diabetes management [30, 31, 34, 39]. One study did not specify daily carbohydrate intake levels for the control group [41].

Regarding changes in daily carbohydrate intake, seven studies documented a substantial reduction in total daily carbohydrate intake of the KD groups [28–31, 36, 37, 41] (Table 5). The range of carbohydrate reduction was from 111.45 g after 6 months [37] to 200 g after 48 weeks [29]. At the final follow-up, the daily carbohydrate intake ranged from 33.8 g at 16 weeks [41] to 85 g at 1 year. Three studies reported changes in daily net carbohydrate intake from baseline and reported a substantial reduction in daily net carbohydrate intake ranging from 102.5 at 12 months to 122.7 at 32 weeks [34].

**Fig. 2:**
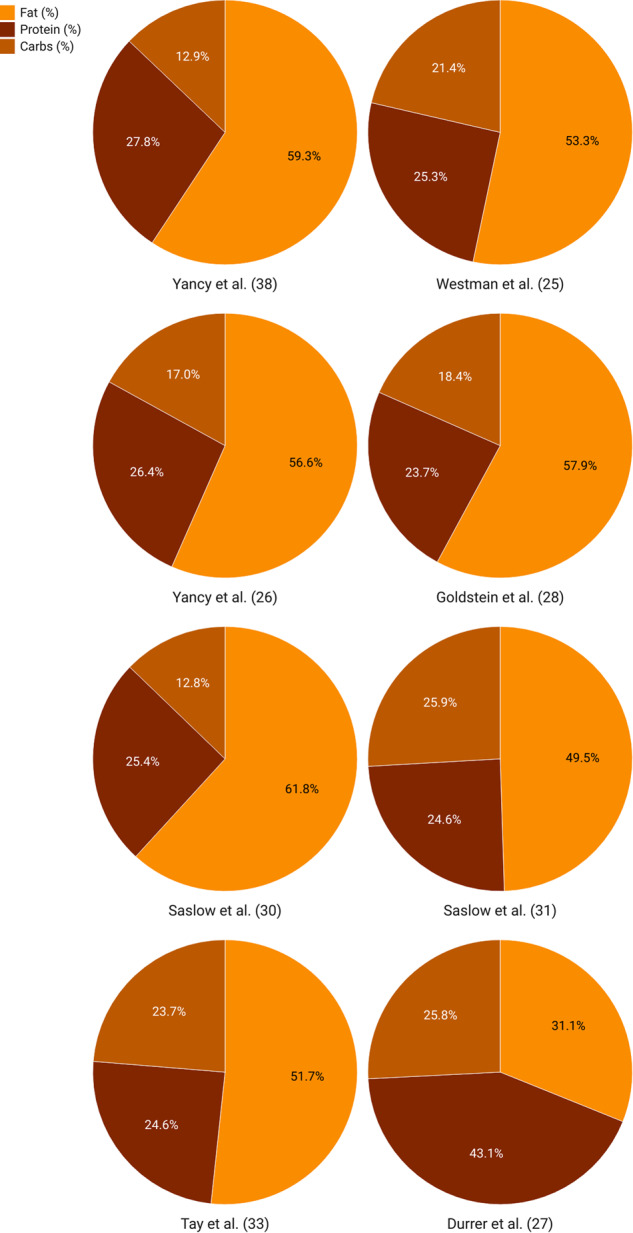
Percentage of macronutrient intake from daily caloric intake.

To standardize the daily carbohydrate intake among included studies, we calculated the percentage of total calories from carbohydrates based on the data obtained from eight studies that measured daily protein, fat, and caloric intake (Fig. 2). We found that the daily percentage of total calories from carbohydrates varied from 12.8% at 12 months [32] to 25.9% at 12 weeks [34]. The control group consumed a normal range of carbohydrate intake that ranged from 38.5% at 12 weeks [30] to 53.8% after 2 years [36]. Finally, among studies that compared carbohydrate intake levels between the KD and control groups, five studies found significantly lower carbohydrate intake in KD compared to the control groups at the final follow-up [29, 31, 32, 34, 36].

### Intervention effect on protein intake

The daily protein intake in the KD group was unrestricted in four studies [28, 29, 31, 41] (Table 3). Three studies advised for an adequate amount of daily protein [33, 37, 39], three studies specified the amount of daily protein intake as a percentage of total daily caloric intake or in grams per day [30, 35, 40]. One study did not recommend the amount of protein intake for the KD group [34]. Regarding the control group, two studies indicated a specific daily protein intake goal [31, 35], and three studies recommended that participants to follow lifestyle guidelines for protein intake [30, 33, 39]. In contrast, three studies did not specify daily protein intake for the control group [28, 29, 34], with one study recommending participants to consume protein from lean protein sources [34].

The changes in daily protein intake in the KD group varied among studies (Table 5). Five studies documented an increase in daily protein intake [28–30, 32, 41], ranging from 2.7 g/d at 16 weeks [41] to 22 g/d at 24 weeks [28]. Two studies reported a very slight reduction in daily protein intake, with Goldstein et al. reporting 3 g/d [31] and Saslow et al. reporting 1.6 g/d reduction [32] at 12 months and at 32 weeks, respectively. The final follow-up daily protein intake in the KD group ranged from 81.7 g/d at 32 weeks [34] to 112.2 g/d at 48 weeks [29]. Out of the 6 studies that measured changes in daily protein intake from baseline to final follow-up in the control group, 5 reported a decrease [28–32, 34] ranging from 0.1 g/d after 32 weeks [34] to 29 g/d after 12 months [31]. In contrast, Durrer et al. reported a slight increase of 3 g/d [30]. The daily protein intake in the control group at the final follow-up ranged from 67 g/d after 24 weeks [28] to 90 g/d after 12 weeks [30]. Three studies compared daily protein intake between the KD and control diet. Two studies found a significantly higher daily protein intake in the KD group compared to the control group [32, 36], while one did not find a difference between the two groups [34].

According to Fig. 2, the participants in the KD group consumed a moderate amount of protein, which ranged from 16.4% after 12 months [32] to 21.6% of total daily calories after 12 weeks [30]. On the other hand, the control group appeared to consume a higher protein intake, ranging from 23.7% after 12 months [31] to 43.1% after 12 weeks [30].

### Intervention effect on fat intake

There were no restrictions on daily fat intake in the KD group in 7 studies [28, 29, 31, 32, 37, 39, 41], whereas 3 studies set a specific initial fat intake goal (Table 3). For example, Tay et al. suggested 58% fat from total calorie intake [35], Walton et al. suggested 70–75% [40], and Durrer et al. suggested fat intake to be 35–45 g/d [30]. The control group in 5 studies did not have a specific daily fat intake but were advised to follow diabetes management guidelines on a low-fat eating pattern [28, 30, 33, 34, 39]. Two studies recommended less than 30% of fat from total daily calorie intake for the control groups [29, 35] whereas Goldstein et al. suggested dividing 80% of daily caloric intake between fat and carbohydrates [31].

In 3 studies, daily fat intake increased from baseline to final follow-up [28, 29, 32], with an increment ranging from 1.8 g/d after 48 weeks [29] to 26.2 g/d after 12 months [32] (Table 5). Conversely, in 3 studies, a decrease in daily fat intake was reported from baseline to final follow-up ranging from a reduction of 1 g/d after 12 months [31] to 38 g/d after 12 weeks [30]. At the final follow-up, the daily fat intake ranged from 34 g/d at 12 weeks [30] to 111 g/d at 12 months [31]. All 6 studies that assessed changes in daily fat intake from baseline in the control group observed a reduction [28–32, 34], ranging from 2 g/d after 12 weeks [30] to 43 g/d after 12 months [31]. At final follow-up, daily fat intake in the control group ranged from 55 g/d after 24 weeks [28] to 85 g/d after 12 months [31]. Among the 4 studies that compared fat intake between the KD and control group, 3 reported significantly higher fat intake in the KD compared to the control group [31, 32, 36], while one reported no significant difference in daily fat intake between the 2 intervention groups [34].

In general, KD groups showed a greater proportion of their calorie intake from fat. All but 1 study [30] reported that the percentage of daily caloric intake from fat was greater than 50% in the KD group, while in the control group was at or below 40%. Total daily fat intake ranged from 31.1% [30] to 61.8% [34] in the KD group, compared to 28.2% [36] to 40.4% [32] in the control group.

### Intervention effect on ketone level

The definitions of adherence to the KD based on ketone levels varied across studies (Table 6).

**Table 6:** Changes in ketone and physical activity levels across included studies.

Author	Ketone		Physical activity
	Adherent definition	Adherent outcomes	Findings
Yancy [38]	Urine ketone level >0 mmol/L	% adherent participants at week 16: 9.5%	N.R.
Tay [32, 33]	Plasma ketone concentration	N.R.	No difference between groups on changes in activity levels and exercise session attendance at weeks 52 and 2 years (*p* > 0.05)
Goldstein [28]	Urine ketone >0 mmol/L	% adherent participants at 1 year: 65%	Both diet groups similarly increased their reported exercise activity during the trial by ~1 h/week at 1 year
Westman [25]	Urine ketone concentration	N.R.	No difference between groups on PA levels at 24 weeks
Yancy [26]	Urine ketone concentration ≥0.9 mmol/L	% adherent participants at 48 weeks: 13%	No difference between groups on PA levels at 48 weeks
Saslow [29, 30]	Self-measured finger-stick BHB concentration between 0.5–3 mmol/L	N.R.	No changes in PA within the group and no differences between groups on PA levels at 1 year
Saslow [31]	Self-measured urine acetoacetate >0	N.R.	N.R.
Durrer [27]	Cpillary blood ketones	N.R.	No difference between groups on PA levels at 12 weeks
Mason [34]	Self-measured finger-stick BHB concentration ≥0.3 mmol/L	N.R.	N.R.
Walton [37]	Plasma ketone concentration ≥0.5 mmol/L	Mean (SD) plasma ketone concentration (mmol/L) at 12 weeks: 1.3 (0.15)	N.R.
Hallberg [36] Athinarayanan [35]	Finger-stick or lab-measured plasma BHB concentration between 0.5–3 mmol/L	Between day 0 to 1 year, 96% of completers reported at least one BHB > 0.5 at 1 year, 61.5% uploaded at least one BHB > 0.5 between 1 and 2 years Mean (SD) lab-measured ketone concentration (mmol/L) at 2 year: 0.18 (0.04)	N.R.

*NR* not reported, *BHB* beta-hydroxybutyrate, *PA* physical activity.

Four studies used the presence of ketones to define KD adherence [31, 34, 36, 40], with higher levels of ketone concentrations indicating better adherence, while five studies defined adherence as meeting a specific threshold of ketone levels [29, 32, 37, 39, 41]. Specific thresholds included urine ketone levels ≥0.9 mmol/L (*n* = 2) [29, 41], plasma BHB levels between 0.5 to 3 mmol/L (*n* = 2) [33, 39], and plasma BHB ≥ 0.3 mmol/L (*n* = 1). Two studies did not provide information on how adherence was defined based on ketone levels [37].

Depending on the criteria used to determine adherence and how it was reported, the percentage of participants who adhered to the KD varied across studies, ranging from 9.5% at 16 weeks [41] to 98% at 1 year [39] (Table 6). For example, Yancy et al. reported that only 2 out of 21 participants (9.5%) had urine ketone levels ≥0.9 mmol/L at the 16-week follow-up visit, whereas Hallberg et al. reported adherence as the percentage of study completers who reported at least one home-monitored blood BHB ≥ 0.5 mmol/L during year 1 and year 2.

Two studies measured changes in plasma ketone concentrations in participants following the KD and observed an increase in plasma ketone levels during the intervention compared to baseline [38–40]. Hallberg et al. and Athinarayanan et al. reported an increase in plasma BHB from 0.17 mmol/L at baseline to 0.31 mmol/L at year 1 and 0.27 mmol/L at 2 years. Similarly, Walton et al. found that the average plasma ketone level increased from 0.9 mmol/L at week 1 to 1.3 mmol/L at week 12.

### Intervention effect on physical activity

All included studies provided lifestyle education on PA or encouraged PA during the intervention, with one study including supervised exercise sessions [35] (Table 3). Five studies compared PA levels between the KD and control groups but found no significant differences in PA levels between groups [28–30, 32, 36], despite various methods that were used to assess PA levels (Table 6).

## Discussion

A low level of adherence to the KD prevents valid assessment of the efficacy of the diet and may limit its effect on weight and T2D management [20]. However, to date, no studies have systematically examined the assessment methods used to measure diet and PA adherence levels in lifestyle interventions with KD or the extent to which patients could adhere to the KD regimen. To address this knowledge gap, we conducted a scoping review to map out the various methods used to evaluate KD adherence and PA levels and to report on the macronutrient intakes in patients under the KD and the levels of PA in studies that utilized KD as part of a lifestyle intervention among adults with overweight/obesity and T2D. Our findings revealed substantial heterogeneity in the methods used for measuring and reporting diet and PA levels in these interventions. Notably, there was great variability in carbohydrate intake, with most studies reporting daily consumption levels exceeding the recommended amount for the KD [7]. Furthermore, the studies reported a moderate level of protein intake, and a lower amount of fat intake than the recommended amount for KD [7]. We also found that total caloric intake was slightly restricted in some of the studies.

The methods for assessing adherence to the KD varied greatly across the included studies. Self-reported methods such as 24-hour dietary recall and dietary records are commonly used for assessing diet adherence in patients with T2D and can be extended to diverse eating patterns [42]. Subjective dietary records can provide a snapshot of a patients’ eating habits, enabling health professionals to offer recommendations regarding dietary modifications. However, a key limitation of self-reported methods is the possibility of underestimation. Previous studies have revealed that self-reported dietary records could underestimate daily energy intake by 23% among adults [43], as well as the absolute intake of fat, protein, and carbohydrates [44].

On the other hand, ketone levels represent an objective biomarker for determining KD adherence. The KD stimulates the synthesis of ketone bodies in the liver as an energy source, resulting in elevated levels of circulating ketones in the blood and urine [45]. Using ketone levels as a means of assessing adherence is advantageous by overcoming errors and recall bias of subjective measures [43]. However, in this study, we were unable to investigate and compare changes in ketone levels across studies due to great variability in the methods used for assessing ketones and only 1 study reported changes in BHB levels from baseline to follow-up visits. Hence, to improve the evaluation and comparison of ketone-indicated diet adherence levels, it is necessary for future studies to establish standardized protocols for assessing ketones, including what ketone bodies to measure, when to measure them, and the reporting of changes in ketone levels before-and-after the diet. Furthermore, as there is no definitive “gold standard” for assessing diet adherence, future research may consider incorporating both ketone measures and self-reported dietary records to provide a comprehensive reflection of adherence to the KD [46].

Our findings are consistent with previous research indicating that adherence to the self-prepared KD is low [47]. Despite the implementation of various behavior change techniques to improve adherence to the KD, we found that reducing carbohydrate intake and increasing fat consumption was especially challenging for this population. A personalized nutrition recommendation system could be a solution to enhance adherence to the KD, in which machine learning algorithms are used to generate personalized meal plans and recipes for patients to follow, based on a range of factors, including but not limited to dietary preferences, nutritional requirements, personal characteristics, and vital signs [48]. For example, Sookrah et al. developed a diet recommendation system that produced customized meal plans and recipes conforming to the DASH diet for hypertension patients [49]. The system factored in diverse criteria, including allergies, blood pressure level, age, weight, smoke/alcohol intake, dietary intake, and food preferences. They found that the diet recommendation system was highly accepted and deemed feasible in assisting patients in adhering to the DASH diet and managing blood pressure. On the other hand, studies have indicated that there might be inter-individual variabilities in the metabolic responses to the same food and macronutrient proportions [50, 51]. Therefore, developing machine learning-based diet recommendation systems that consider the unique needs of adults with overweight/obesity and T2D and their inter-personal variabilities on metabolic responses could facilitate the effective translation of the KD on T2D management, improve adherence to the KD, induce ketosis, and ultimately improve population health.

Interestingly, our analysis revealed that the caloric intake was comparable between the KD group and the control group, as well as other lifestyle interventions with varying macronutrient compositions [52]. Meanwhile, our findings are consistent with prior studies indicating that the KD led to better weight loss and HbA1c control compared to other types of diet [15, 18]. A possible explanation for the promising T2D management observed in the KD group could be the increased energy expenditure. Previous research has suggested that adults with overweight/obesity following the KD experienced higher energy expenditure compared to other diets [53, 54]. For example, Ebbeling and colleagues found that the KD increased total energy expenditure by ~300 kcal/day and resting energy expenditure by ~67 kcal/day compared to the low-fat diet in adults with overweight/obesity. Thus, further investigation is warranted to explore the impact of the KD on energy expenditure in adults with overweight/obesity and T2D [54].

The greater improvement in weight loss and diabetes management observed in the KD group could also be attributed to the changes PA levels. Previous research has found that KD could improve physical function, reduce fatigue, and increase perceived energy levels in women with ovarian or endometrial cancer after 12 weeks [55]. Additionally, research comparing the KD and low-fat diets in breast cancer patients found that the KD group reported higher levels of physical activity after 6 weeks [56]. However, we found that none of the included studies reported significant differences in PA levels between diet groups. Nevertheless, there were great inconsistencies regarding the assessment tools used to evaluate PA, and a large proportion of studies relied on self-reported PA measurements that were subject to recall and response bias, potentially compromising the accuracy of PA evaluation [57]. Therefore, further research is necessary to examine changes in PA levels using more accurate assessment tools, such as accelerometers and pedometers, to determine whether changes in PA levels could account for better weight loss and diabetes management in the KD groups.

Surprisingly, none of the included studies specified the ketogenic ratio for the KD group. The ketogenic ratio is defined as the grams of fat to the grams of carbohydrate plus protein [58]. A minimum ketogenic ratio of 1.5 is necessary to achieve ketosis, with higher ratios associated with higher ketone levels [21]. In this review, we found higher carbohydrate and lower fat intakes compared to the recommended, indicating a lower ketogenic ratio, which could lead to a decreased level of ketosis. The ketogenic ratio plays a crucial role in determining the efficacy and tolerability of a KD. While studies have identified a positive association between a higher ketogenic ratio and better health outcomes, such as seizure control, weight management, and risk for diabetes [59, 60], the strict dietary regimen required to achieve a higher ratio may pose KD adherence challenges [58]. As the effects of different levels of ketogenic ratio on weight and T2D management remain inconclusive, it is imperative to investigate how people living with overweight/obesity and T2D respond to varying ketogenic ratios and determine the optimal KD based on health outcomes and its tolerability.

There are several key strengths of our review. First, we examined KD adherence based on both objective and subjective methods, providing a thorough understanding of low adherence within the population. Second, by excluding feeding-controlled trials and diet-only trials, we focused on lifestyle interventions that integrate KD for managing obesity and T2D. Considering the increased adoption of KD as part of lifestyle interventions along with PA and/or behavior change strategies, our review offers valuable insights into the real-world level of adherence to KD. Third, we are the first study to describe and compare changes in PA levels following KD interventions in this population. Despite existing evidence emphasizing the importance of PA as a key component of lifestyle interventions and its independent effect on weight management and T2D outcomes, our findings shed light on the necessity of assessing changes in PA in future KD interventions for this population. Lastly, our use of a consistent definition of KD with clear macronutrient cutpoints enhanced the comparability between studies and ensured the reliability of our findings.

This scoping review has several limitations that should be considered. First, most of the included studies were conducted in the United States, which could limit the generalization of the findings to other countries with different eating habits. Second, although all studies included “adults” as participants, the sample was predominantly middle-aged adults, which limited the generalization of intervention findings to adults of other age groups. Third, the included studies varied in study design, intervention delivery mode, and definition for KD. This inherent lack of homogeneity across the included studies made it difficult to perform a meaningful comparison across studies. Fourth, our assessment of the studies was limited to comparing dietary and physical activity levels at “baseline” and “final follow-up”, providing restricted insight into how these levels changed during the intervention. Finally, we did not undertake a quality appraisal of the included studies due to the nature of the scoping review.

## Conclusion

In conclusion, this scoping review aimed to identify the methods used to evaluate KD adherence and PA levels in adults with overweight/obesity and T2D in lifestyle interventions and to examine their levels of macronutrient intake to KD. We observed considerable variability in the methods for assessing diet and PA and found that adherence to the KD was low, particularly due to excessive carbohydrate intake and inadequate fat intake. To improve future interventions, we recommend using the ketogenic ratio to prescribe the KD and adopting machine learning techniques to generate personalized nutrition recommendations that meet the KD requirements. Additionally, standardized approaches and transparent reporting of diet and PA assessments are essential to facilitate their translation into healthcare practice and policy.

### Supplementary information

Appendix 1

## Data Availability

The data generated during and/or analyzed during the current study are available from the corresponding author on reasonable request.
