# Supplementary material for: Adherence to ketogenic diet in lifestyle interventions in adults with overweight or obesity and type 2 diabetes: a scoping review
Source: Nutr Diabetes. 2023 Sep 14;13:16. doi: 10.1038/s41387-023-00246-2 (PMC10502148; doi:10.1038/s41387-023-00246-2)
Supplement: Supplementary file 1 — Appendix 1 [file 41387_2023_246_MOESM1_ESM.docx]

**Appendix 1. Search strings.**

| **Search** | **Query** |
| --- | --- |
| #1 | KETOGENIC DIET – CONCEPT  (((((diet, ketogenic[MeSH Terms]) OR (diet, carbohydrate restricted[MeSH Terms])) OR (ketogenic diet)) OR (modified atkins diet)) OR (((low[TIAB] OR restrict*[TIAB]) AND carb*[TIAB] AND diet*[TIAB]))) OR (((low[TIAB] OR restrict*[TIAB]) AND sugar[TIAB] AND diet*[TIAB])) |
| #2 | DIABETES TYPE 2 – CONCEPT  "Diabetes Mellitus, type 2"[MeSH Terms] OR "type 2 diabetes mellitus"[TIAB] OR "type 2 diabetes"[TIAB] OR "type II diabetes"[TIAB] OR T2D[TIAB] OR T2D[OT] OR T2DM[TIAB] OR NIDDM[TIAB] OR MODY[TIAB] OR MODY[OT] OR (((adult[TIAB] OR mature[TIAB] OR maturity[TIAB]) AND onset[TIAB]) AND (diabetes[TIAB] OR diabetic[TIAB])) |
| #3 | #1 AND #2 |
| Limited to date (2005) | |
| Limited to English | |
